# Do they really support “your freedom of choice”? FoPNL and the food industry in Brazil

**DOI:** 10.3389/fnut.2022.921498

**Published:** 2023-01-19

**Authors:** Laís Amaral Mais, Mélissa Mialon, Bruna Kulik Hassan, João Marcos Darre Peres, Mariana Gondo dos Santos, Ana Paula Bortoletto Martins, Janine Giuberti Coutinho, Camila Maranha Paes de Carvalho

**Affiliations:** ^1^Instituto Brasileiro de Defesa do Consumidor (IDEC), São Paulo, Brazil; ^2^Núcleo de Pesquisas Epidemiológicas em Nutrição e Saúde (NUPENS), Universidade de São Paulo (USP), São Paulo, Brazil; ^3^Trinity College Dublin, The University of Dublin, Dublin, Ireland; ^4^ACT Promoção da Saúde, São Paulo, Brazil; ^5^Universidade Federal Fluminense (UFF), Rio de Janeiro, Brazil; ^6^O Joio e o Trigo, São Paulo, Brazil

**Keywords:** front-of-package labeling, nutrition labeling, corporate political activity, conflict of interest, food regulation

## Abstract

**Introduction:**

In 2020, Brazil approved the introduction of a new front-of-package nutrition labeling (FoPNL) in the format of a magnifying glass (MG) after years of discussion. There is currently a lack of understanding of the role of the food industry in that process. This study aimed to describe the corporate political activity (CPA) of the food industry and conflicts of interest situations, as they happened during the development and approval of a new FoPNL system in Brazil.

**Materials and methods:**

We undertook bibliographical and documentary searches using material from food companies, trade associations and front groups involved in the regulatory process. We (1) collected information about the case study context, (2) collected data from documentary sources, and (3) prepared a synthesis of the results and a timeline of key events.

**Results/Discussion:**

During the FoPNL regulatory process in Brazil, the food industry opposed the introduction of warning labels, a model supported by health authorities and implemented with success in other countries in Latin America. The food industry rather promoted a traffic-light labeling system, known to be less effective at guiding individuals to make healthier food choices. Later in the process, when it was evident that its preferred model would not be used, and a MG would rather be introduced, the food industry argued for the use of a different version of this FoPNL model. We found that the food industry, all along the process, was directly involved in and influenced the development of the FoPNL, by providing technical support, advising and lobbying policymakers. The food industry also established relationships with a consumer non-governmental organization and nutrition professional societies. The food industry also produced and disseminated information supporting its position in order to influence public opinion and high-level decision makers, and used the legal system to delay the process.

**Conclusion:**

The FoPNL in Brazil is neither aligned with the recommendations of international health organizations nor with existing independent scientific evidence. The new FoPNL, as adopted in Brazil, reflects some of the preferences of the industry; it is likely that the influence of that sector during the legislative process was pivotal, even if its initial proposal was not adopted.

## Introduction

The implementation of a front-of-packageprotect nutrition labeling (FoPNL) on food products is a key element that could help promote and protect health and health-related rights (1), and is recommended by the World Health Organization (WHO) (2) and the United Nations (UN) Special Rapporteur on the right to health (3). Clearer nutrition labels, as proposed with the introduction of FoPNL, have the potential to increase people’s understanding of the healthiness of food products, thus helping them shift to healthier options (4–6). Clearer FoPNL, therefore, contributes to the reduction in the consumption of foods that contain too much sugar, fats and sodium, particularly ultra-processed food products, and is a cost-effective option for the control and reduction of non-communicable diseases (NCDs) (7). FoPNL is considered a cost-effective “stepping stone” for other measures that promote healthy diets, such as taxation, school environment regulation, and marketing restrictions (1, 3).

Several countries around the world have adopted FoPNL systems (8). Warning labels (WLs) of a geometric form, such as an octagon, on products with an excessive amount of nutrients of concern, are now used in Latin American countries (Chile, Peru, Uruguay, and Mexico; and in the process to be implemented in Argentina and Colombia). Comparative studies have shown that WLs are the most effective FoPNL system for the identification of unhealthy food products (9–12). Indeed, 1 year after the implementation of the Chilean FoPNL, a study showed a significant decrease in the consumption of food products that contain too much added sugar and sodium (13). In Uruguay, 58% of individuals surveyed reported modifying their purchasing decisions for healthier products after having seen WLs, only 1 month after the implementation of a FoPNL system (14).

Brazil was the first Latin American country to implement a nutrition food labeling regulation in 2003 (15). Since 2014, the Brazilian Health Surveillance Agency (*Agência Nacional de Vigilância Sanitária*–Anvisa) has led a discussion for the update of that regulation, in light of new scientific evidence and international recommendations. The process was carried out with representatives of civil society organizations, academia, the government and the food and beverage industry. In October 2020, a new regulation on nutrition labeling of packaged foods was approved (16, 17), with the inclusion of a mandatory FoPNL, using a magnifying glass (MG) for those products exceeding sets levels of added sugar, saturated fat and/or sodium. The implementation of the norm will go into effect in October 2022.

That process was not necessarily a smooth one. Large food manufacturers make a huge profit from the sales of their products, and the industry’s profits are higher for unhealthy products, such as those high in salt, sugar and fat (18). A FoPNL that would limit the sales of those unhealthy products would direct individuals to healthier, but usually less profitable, choices. In Brazil, trade associations and the food industry therefore tried to influence the regulatory process and public opinion during the early development of the new FoPNL in the country (19). The industry used arguments that delegitimized Anvisa’s role in the process, justified the involvement of the food industry, and shifted the blame away from the role of unhealthy food products for ill-health at the population level, and instead focused on individuals as responsible for their choices. These arguments from the industry were mostly based on an inconsistent set of non-academic and non-peer-reviewed evidence (20). Other countries in Latin America also faced strong opposition from the food industry to the introduction of new FoPNL systems (21–23). In Brazil, beyond these arguments used in the early stages of the regulatory process, the specific actions of the food industry to influence the entire process have not yet been explored.

The aim of the present study was therefore to identify the main events during the regulatory process for a new FoPNL in Brazil, and to map the actions used by the food industry, also known as corporate political activity (CPA), and the conflicts of interest (CoI) involved in that process (21, 24). The CPA of the food industry refers to action-based strategies (coalition management, information management, direct involvement and influence in public policy, legal action) and argument-based strategies (e.g., questioning the governance of a process; promoting solutions to health-related issues that have no evidence of their effectiveness, etc.) (21). CPA occurs through an action or argument used between two parties: the food industry and a third party (e.g., a policymaker, or a researcher). A CoI, as per the law in Brazil, refers to the case where a person, in a position or employment at the federal Executive Branch: (a) disclose or make use of privileged information, for one’s own benefit or that of a third party; (b) carry out activities that imply the provision of services or the maintenance of a business relationship with an individual or legal entity that has an interest in a decision of the public agent or of a collegiate in which he participates; (c) carry out an activity that is incompatible with the attributions of the position or job; (d) act, even if informally, as attorney, consultant, advisor or intermediary of private interests in the bodies or entities of any of the Branches of the Union, the states, the Federal District and the municipalities; (e) perform an act for the benefit of the interest of a legal entity in which the public agent, his/her spouse, partner or relatives, by blood or related, in a direct or collateral line, up to the third degree, and which may benefit or influence in its management acts; (f) receive gifts from those who are interested in a decision by the public agent or collegiate in which he or she participates outside the limits and conditions; (g) provide services, even if occasional, to the company whose activity is controlled, inspected or regulated by the entity to which the public agent is linked (25).

## Materials and methods

This study was of a qualitative nature. We undertook bibliographical and documentary searches, based on a combination of a case study design with a constructivist approach to the policy analysis methodology (26). Data collection and analysis were conducted between July and December 2021 by LAM with support from the other authors. All the authors contributed to the review and analysis of the data collected. The research team has expertise in public health nutrition, food policy and in industry interference in Brazil, from academia, civil society and the media. All but one author (MM) of the present study, through their work in academia, civil society and the media, were directly involved in the regulatory process under study. While our collective experience can provide crucial knowledge on the subject, it also meant that we took a critical approach to the actions described below. This work was therefore aligned with a critical social science perspective (27).

The searches conducted involved three main steps. First, we searched for information about the context of the case study (Brazil, Federal level). Second, we collected data from documentary sources. Finally, a synthesis of the results and a timeline of important CPA of the food industry during the regulatory process of the nutrition labeling of packaged foods regulation were elaborated.

The time period under study started in 2011, when Brazil officially requested the Southern Common Market (MERCOSUR) a review of the general and nutrition labeling on food products, until April 2022, when this article was finished. This represents the entire period of technical discussions and Anvisa’s regulatory process of nutrition labeling of packaged foods, including the approval of the new regulation, but not the beginning of its implementation, which is planned to happen in October 2022.

The food industry included, for our analysis: food companies and trade associations directly involved in the regulatory process and the Labeling Network (*Rede Rotulagem*, in Portuguese), a group founded by several of these actors from the industry during this period (28).

Documentary searches were conducted using multiple sources. Documents from industries involved in the process were searched on the websites and social media profiles of the industry actors that were members of *Rede Rotulagem* (28), Your Freedom of Choice (*Sua Liberdade de Escolha*, in Portuguese) (29) and Eye on the MG (*Olho na Lupa*, in Portuguese) (30), such as the Brazilian Association of the Food Industry (*Associação Brasileira da Indústria de Alimentos*–ABIA) (31), the Brazilian Association of Soft Drinks and Non-Alcoholic Beverage Industries (*Associação Brasileira das Indústrias de Refrigerantes e de Bebidas não Alcoólicas*–ABIR) (32) and the Brazilian Dairy Association (*Associação Brasileira de Laticínios*–*Viva Lácteos*) (33). Official publications from Anvisa on the regulatory process, such as technical reports, presentations, regulations and standards were identified on its official website (34), as well as Brazilian Federal laws (35). Press and media articles were identified using Google searches. Google Scholar was used for identifying publications that have discussed the role of the food industry in the regulatory process in Brazil. A copy of some files of the regulatory process and other documents were obtained via the Access to Information Law (*Lei de Acesso à Informação*–LAI). Requests were made directly to Anvisa, on different occasions between 2017 and 2021. The requests focused on minutes of meetings of Anvisa working groups on labeling from 2011 to 2016; minutes of meetings between directors and corporations or trade associations; minutes of meetings between directors and researchers or research associations with potential CoI; access to documents submitted by corporations or trade associations to the General-Management of Foods (*Gerência-Geral de Alimentos*–GGALI) of Anvisa. Searches were also based on our collective expert knowledge of the case under study.

A distinction was made between independent sources and those from the food industry (as per the affiliations of authors, or as declared in the funding or CoI sections of publications, for example), in order to identify any CoI which may have led to bias.

Data was triangulated amongst those sources and we continued with our searches until no new data was identified.

The analysis and report of the material gathered included: (i) a description of the context under study and a timeline of key events; (ii) a reporting of the CPA of the food industry, with a particular focus on key events, identified as moments that had relevant importance in the regulatory process that were emphasized and mentioned by the food industry actors or by other representatives involved in the process. For that analysis, we used a deductive approach, using the existing classification of CPA described by Mialon et al. (21). Findings were synthesized narratively, using illustrative examples of the practices used by the industry in Brazil.

The investigation was funded by Bloomberg Philanthropies (grant number BRAZIL-RIIO-05B). The study funder played no role in any stage of the research. Interpretation of the data and findings are from the authors alone.

The study did not require ethics approval, since only secondary and publicly available data was used.

## Results

Table 1 is a timeline of key CPA strategies and CoI situations that we have documented for the regulatory process during the development of a new FoPNL in Brazil. Table 2 presents the main actors from the industry, academia, civil society and government involved in that process.

**TABLE 1:** Timeline and classification of the main corporate political activities and conflicts of interest situations during the regulatory process of nutrition labeling of packaged foods.

Date	Event	Classification of the CPA and CoI events			
		Strategies		Practices/Domain	Mechanisms/Arguments
June 2017	Creation of the *Rede Rotulagem*	Instrumental	Coalition management	Establish relationships with key opinion leaders and health organizations	–Promote public-private interactions with health organizations –Establish informal relationships with key opinion leaders
				Establish relationships with the media	–Establish close relationships with media organizations, journalists and bloggers to facilitate media advocacy
November 2017	Publication of the opinion poll “Population disposition for change in labeling of foods and non-alcoholic beverages categories” by IBOPE, commissioned by ABIA	Instrumental	Information management	Production	–Fund research, including through academics, ghost writers, own research institutions and front groups
				Amplification	–Cherry pick data that favors the industry, including use of non-peer reviewed or unpublished evidence
			Direct involvement and influence in policy	Actor in government decision making	–Provide technical support and advice to policymakers (including consultation)
		Discursive		Frame the debate on diet- and public health-related issues	–Shift the blame away from the food industry and its products, e.g., focus on individual responsibility, role of parents, physical inactivity
July 2018	Presentation of a lawsuit by ABIA requesting the extension of the TPS	Instrumental	Legal action	Use legal action (or the threat thereof) against public policies or opponents	–Litigate or threaten to litigate against governments, organizations or individuals
July 2018	Publication of the economic study “Socioeconomic impacts of the implementation of nutrition labeling models on the front panels of foods and beverages” by GO Associados, commissioned by the *Rede Rotulagem*	Instrumental	Information management	Production	–Fund research, including through academics, ghost writers, own research institutions and front groups
				Amplification	–Cherry pick data that favors the industry, including use of non-peer reviewed or unpublished evidence
		Discursive		The economy	–Stress the number of jobs supported and the money generated for the economy
				Expected food industry costs	–Policy will lead to reduced sales/jobs –Cost of compliance will be high
September 2018	Lobby with the former President of the Republic, Michel Temer, for the nomination of the former Director-President of Anvisa, William Dib	Instrumental	Direct involvement and influence in policy	Indirect access	–Lobby directly and indirectly (through third parties) to influence legislation and regulation so that it is favorable to the industry
November 2019	Request of ABPA to Anvisa for the extension of the PCs	Instrumental	Direct involvement and influence in policy	Indirect access	–Lobby directly and indirectly (through third parties) to influence legislation and regulation so that it is favorable to the industry
January–December 2019	Meetings of the leaders of Anvisa with the private food sector	Instrumental	Direct involvement and influence in policy	Indirect access	–Lobby directly and indirectly (through third parties) to influence legislation and regulation so that it is favorable to the industry
				Actor in government decision making	–Provide technical support and advice to policymakers (including consultation)
June 2020	Presentation of a letter of the Embassy of Italy in Brazil to the	Instrumental	Coalition management	Constituency fabrication	–Procure the support of community and business groups to oppose public health
	Presidency of the Republic and to Anvisa about the concerns of the agri-food sector Italian companies about the approval and implementation of a FoPNL in warning format			Constituency fabrication	– measures
		Discursive		Expected food industry costs	–Policy will lead to reduced sales/jobs –Cost of compliance will be high
				Frame the debate on diet- and public health-related issues	–Promote industry ìs preferred solutions: education, balanced diets, information, public private initiatives, self-regulation (reformulation)
December 2020	Interview of the former General-Manager of Foods of GGALI/Anvisa, Thalita Lima, to *ILSI em foco*	Instrumental	Coalition management	Establish relationships with key opinion leaders and health organizations	–Establish informal relationships with key opinion leaders
			Direct involvement and influence in policy	Indirect access	–Lobby directly and indirectly (through third parties) to influence legislation and regulation so that it is favorable to the industry
		Discursive		Frame the debate on diet- and public health-related issues	–Stress the good traits of the food industry
July 2021	Revolving doors of the former Director of Anvisa and rapporteur of the regulatory process of nutrition labeling of packaged foods, Alessandra Soares	Instrumental	Direct involvement and influence in policy	Indirect access	–Use the “revolving door,” i.e., ex-food industry staff work in government organizations and vice versa
September 2021	Publication of the paper “Comparison of the efficacy of five front-of-pack nutrition labels in helping the Brazilian consumer make a healthier choice” by Unilever employees	Instrumental	Information management	Production	–Fund research, including through academics, ghost writers, own research institutions and front groups
				Amplification	–Cherry pick data that favors the industry, including use of non-peer reviewed or unpublished evidence
March 2022	Creation of *Olho na Lupa*	Instrumental	Coalition management	Establish relationships with the media	–Establish close relationships with media organizations, journalists and bloggers to facilitate media advocacy
			Information management	Amplification	–Cherry pick data that favors the industry, including use of non-peer reviewed or unpublished evidence
		Discursive		Frame the debate on diet- and public health-related issues	–Promote industry ìs preferred solutions: education, balanced diets, information, public private initiatives, self-regulation (reformulation)
Throughout the regulatory process	Participation of Anvisa’s employees in events sponsored by the food industry	Instrumental	Coalition management	Establish relationships with key opinion leaders and health organizations	–Establish informal relationships with key opinion leaders
				Establish relationships with the media	–Establish close relationships with media organizations, journalists and bloggers to facilitate media advocacy
			Direct involvement and influence in policy	Indirect access	–Lobby directly and indirectly (through third parties) to influence legislation and regulation so that it is favorable to the industry

Brazil, 2011–2022. Lines in gray refers to conflicts of interest situations. ABIA, Brazilian Association of the Food Industry; ABPA, Brazilian Association of Animal Protein; Anvisa, Brazilian Health Surveillance Agency; CoI, conflicts of interest; PC, public consultation; CPA, corporate political activity; FoPNL, front-of-package nutrition labeling; GGALI, Food General Management; IBOPE, Brazilian Institute of Public Opinion and Statistics; ILSI, International Life Sciences Institute; TPS, Public Collection of Information.

**TABLE 2:** Actors involved in the regulatory process of nutrition labeling of packaged foods.

Name (in Portuguese)	Name (in English)	Abbreviation	Sector*	Description
Agência Nacional de Vigilância Sanitária	Brazilian Health Surveillance Agency	Anvisa	Government	Regulatory agency linked to the Ministry of Health and responsible for the elaboration of rules on food labeling.
Alessandra Bastos Soares	–	–	Government	Director of Anvisa from 2018 to 2020 (final period of the regulatory process) and rapporteur of the regulatory process on nutrition labeling on packaged foods.
Aliança pela Alimentação Adequada e Saudável	Alliance for Adequate and Healthy Diets	–	Civil society	Coalition that brings together 72 collectives, including civil society organizations, social movements, and professional entities, and is currently composed of 350 individual members in defense of the public interest of the defense of the Human Right to Adequate Food.
Associação Brasileira da Indústria de Alimentos	Brazilian Association of the Food Industry	ABIA	Industry	Founded in 1963, it is the majorst association of representatives of the food industry in the country. It brings together small, medium and large industries throughout the national territory, including Brazilian and multinational companies. It is also a member of the Labeling Network.
Associação Brasileira das Indústrias de Refrigerantes e de Bebidas não Alcoólicas	Brazilian Association of Soft Drinks and Non-Alcoholic Beverage Industries	ABIR	Industry	Founded in 1950, it brings together companies that manufacture various non-alcoholic beverages, such as soft drinks, juices, nectars, soft drinks, chocolate drinks, teas, isotonic drinks, energy drinks and mineral waters. It is also a member of the Labeling Network.
Associação Brasileira de Defesa do Consumidor	Brazilian Association for Consumer Defense	PROTESTE	Civil society	Founded in 2001, it is a non-profit association that works with suppliers and authorities to defend the interests of consumers.
Associação Brasileira de Laticínios	Brazilian Dairy Association	Viva Lácteos	Industry	Association that represents the dairy industry and brings together 38 of the main manufacturers and associations of the sector in Brazil. Together, Viva Lácteos associates are responsible for 70% of the production of milk and dairy products in the country. It is also a member of the Labeling Network.
Associação Brasileira de Nutrologia	Brazilian Association of Nutrology	ABRAN	Civil society	Founded in 1973, it is an association that brings together doctors working in the field of nutrology.
Associação Brasileira de Proteína Animal	Brazilian Association of Animal Protein	ABPA	Industry	National association that represents the poultry and pork meats sectors in Brazil. It is also a member of the Labeling Network.
Associação Brasileira de Supermercados	Brazilian Supermarket Association	ABRAS	Industry	Founded in 1968, it brings together representatives of the supermarket sector in the country. It is also a member of the Labeling Network.
Confederação Nacional da Indústria	National Industry Confederation	CNI	Industry	Created in 1938, it is the highest organization of the Brazilian industrial sector. It coordinates a system made up of 27 industry federations from the states and the Federal District–to which more than a thousand employers’ unions are affiliated. It is also a member of the Labeling Network.
Conselho Nacional de Segurança Alimentar e Nutricional	National Food and Nutrition Security Council	Consea	Government	Council for the articulation between government and civil society in proposing guidelines for actions in the area of food and nutrition. The Council has an advisory role and advises the President of the Republic in formulating policies and defining guidelines for the country to guarantee the human right to food. Consea was dissolved in 2019 by President Bolsonaro.
–	Embassy of Italy in Brazil	–	Government	The main Italian diplomatic representation in Brazil.
Empresa Brasileira de Pesquisa Agropecuária	Brazilian Agricultural Research Corporation	Embrapa	Government	Created in 1973, it is a public research company linked to MAPA of Brazil.
Instituto Brasileiro de Defesa do Consumidor	Brazilian Institute for Consumer Defense	Idec	Civil society	Founded in 1987, it is a non-profit consumer association, independent of companies, parties or
				governments. In 2017, Idec presented a proposal to Anvisa to update and improve the nutrition labeling norm in the country. The proposal, based on the PAHO nutrient profile, included adherence to a front-of-package warning labeling model.
–	International Life Sciences Institute Brazil	ILSI Brasil	Industry	Created in 1990, it brings together scientists in the areas of nutrition, biotechnology and risk assessment. In Brazil, the members are companies in the food, agricultural, pharmaceutical and biotechnology sectors.
Jarbas Barbosa	–	–	Government	Director of Anvisa between 2015 and 2018 (period of progress of the regulatory process).
Michel Temer	–	–	Government	Vice President of Brazil from 2011 to 2016. After the impeachment of Dilma Roussef, he took over as president from 2016 to 2019.
Núcleo de Pesquisas Epidemiológicas em Nutrição e Saúde/Universidade de São Paulo	Center for Epidemiological Research in Nutrition and Health/University of São Paulo	Nupens/USP	Academia	It is an integration body of the USP created in 1990 with the purpose of stimulating and developing population research in nutrition and health. The group is composed of professors and researchers, masters, doctoral students and scholarship interns.
Olho na Lupa	Eye on the Magnifying Glass	–	Industry	An initiative of 11 associations representing the food and beverage industry and retail, with the aim to explain how to read and understand the information on food labels to consumers.
Rede Rotulagem	Labeling Network	–	Industry	It is an initiative of the food and beverage production sector that works on the subject of food labeling. It comprises 21 entities, including the industrial and commercial sectors.
Sociedade Brasileira de Alimentação e Nutrição	Brazilian Society of Food and Nutrition	SBAN	Civil society	Founded in 1985, it is a non-profit, scientific civil society whose objective is to stimulate and disseminate knowledge in the field of food and nutrition.
Thalita Lima	–	–	Government	General-Manager of Food at Anvisa between 2015 and 2022 (period of progress of the regulatory process).
Unilever	Unilever	–	Industry	British multinational consumer goods company founded in 1929 and based in London, UK. Its products include food, beverages, cleaning products and personal care products.
Universidade de Brasília	University of Brasília	UnB	Academia	Brazilian public higher education institution located in Brasília, in the Federal District, being one of the most important universities in the country.
William Dib	–	–	Government	Director President of Directors of Anvisa between 2016 and 2019 (period of progress of the regulatory process).
Wilson Mello	–	–	Government	Chairman of the Board of Directors of ABIA between 2018 and 2019.

Brazil, 2011–2022. *The industry classification includes all the industries and front groups represented by and related to food and nutrition. MAPA, Ministry of Agriculture, Livestock and Supply; PAHO, Pan American Health Organization.

A revision of the Brazilian regulation for nutrition food labeling of packaged foods was motivated by two events. First, in 2011, Brazil made a request to MERCOSUR for the revision of nutrition and general food labeling regulations in the region (36). It was accepted by other member countries (Paraguay, Uruguay, and Argentina) and marked the advancement of the international agreements on this theme (37). Furthermore, in 2013, the National Food and Nutrition Security Council (*Conselho Nacional de Segurança Alimentar e Nutricional*–Consea) of Brazil, an entity responsible for monitoring food and nutrition security public policies, published its Resolution no 007/2013, where the Council recommended that Anvisa would need to be moving with “the processes for updating and qualifying regulatory proposals for food labeling” (38). In response to these events, Anvisa created a working group (WG) on nutrition food labeling, with the participation of representatives from the government, civil society, academia and food industry (39). The meetings of the WG occurred from December 2014 to April 2016, and focused on the existing legislation and the possible solutions regarding a new nutrition facts panel, as well as nutrition claims and a FoPNL system (19). At the end of this period, members of the WG were invited to send their proposals about nutrition labeling regulation to Anvisa. We have not encountered any document or activity that could be classified as CPA or CoI for that period.

**FIGURE 1:**
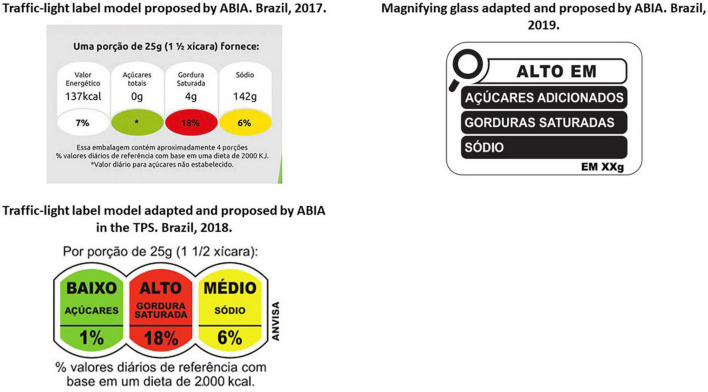
Different FoPNL models proposed by ABIA during the regulatory process. **(A)** Traffic-light label model proposed by ABIA. Brazil. 2017. **(B)** Traffic-light label model adapted and proposed by ABIA in the TPS. Brazil. 2018. **(C)** Magnifying glass adapted and proposed by ABIA. Brazil, 2019. Sources: Anvisa and ABIA. ABIA, Brazilian Association of the Food Industry; TPS, Public Collection of Information.

In 2017, four proposals from WG members were presented to Anvisa: one by the trade association ABIA, two from government bodies, and one from a civil society organization together with a research group from academia. The main disagreement between the proposals was about the FoPNL model (40). While ABIA recommended a traffic-light labeling (TLL) system based on a nutrient profile model (NPM) adapted from the United Kingdom (UK) and supported by the results of an opinion poll about Brazilians’ preference for this model (40, 41) (Figure 1A), the other organizations recommended a WL with a NPM adapted from Pan American Health Organization (PAHO) (42), based on the scientific evidence of its efficacy (10, 43, 44).

In the same period, a group of 21 food industry trade associations, including ABIA, ABIR, *Viva Lácteos*, the Brazilian Supermarket Association (*Associação Brasileira de Supermercados*–ABRAS), the Brazilian Association of Animal Protein (*Associação Brasileira de Proteína Animal*–ABPA), the National Industry Confederation (*Confederação Nacional da Indústria*–CNI), among others, formed a coalition called *Rede Rotulagem* (Tables 1, 2). *Rede Rotulagem* had an official website (28), a newsletter (that started with a weekly periodicity (45, 46), and from May 2019 was then issued on a monthly basis (47, 48) and social media profiles under the name of Your Freedom of Choice (*Sua Liberdade de Escolha*, in Portuguese) (49). This was the CPA strategy that we documented, where the food industry built an internal coalition, for the industry to speak as one, powerful voice, and reached out to key opinion leaders and the media, for building an external coalition that would support and disseminate its position (Table 1).

*Rede Rotulagem* used classic CPA arguments, where it called for free and autonomous food choices, and for the use of the TLL as the clearest and most objective model to inform consumers about nutrition information. *Rede Rotulagem* also rejected the WLs, which were qualified as alarmist and too restrictive compared to the TLL model, which was considered as informative and educational (28, 29, 50). The idea of “fear” and “alarmism” of WLs was further used by ABIA and the food industry.

In November 2017, ABIA commissioned an opinion poll to the Brazilian Institute of Public Opinion and Statistics (*Instituto Brasileiro de Opinião Pública e Estatística*–IBOPE), an example of CPA that could bias the information on which decisions were based. Participants were asked their opinion about WLs in the form of triangles and TLLs. The main result was that the Brazilian population preferred the TLL system over WLs, which were considered as illegible, with incomplete information and associated with fear and guilt (41). ABIA stressed that “everything indicates that this type of alert (WLs) scares more than informs or mobilizes the population.” The results of the poll were disseminated by the food industry (51), especially through *Rede Rotulagem*’s platforms (48) (Table 1).

In December, Anvisa officially launched the regulatory process (52), and a new regulatory impact analysis (*análise de impacto regulatório*–AIR), “a systematic evidence-based regulatory management process that seeks to assess, based on the definition of a regulatory problem, the possible impacts of the options available to achieve the intended objectives” (53).

**FIGURE 2:**
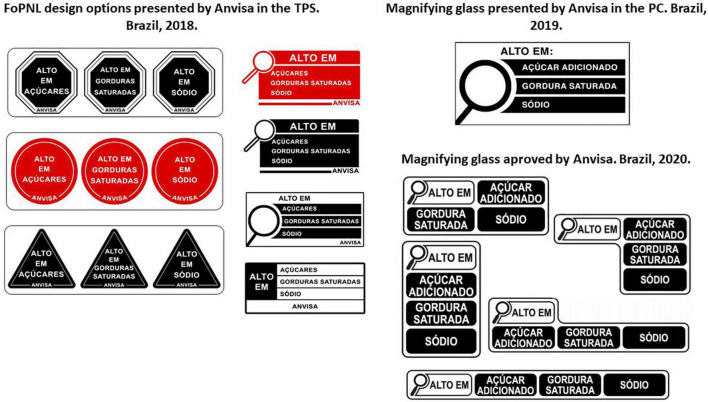
Different FoPNL models proposed by Anvisa during the regulatory process. **(A)** FoPNL design options presented by Anvisa in the TPS. Brazil, 2018. **(B)** Magnifying glass presented by Anvisa in the CP. Brazil, 2019. **(C)** Magnifying glass approved by Anvisa. Brazil. 2020. Source: Anvisa. FoPNL, front-of-package nutrition labeling; Anvisa, Brazilian Health Surveillance Agency; TPS, Public Collection of Information; PC, public consultation.

The first official step in the regulatory process was the Public Collection of Information (*Tomada Pública de Subsídios*–TPS) (54), an online technical consultation based on the Preliminary Regulatory Impact Analysis Report on Nutrition Food Labeling (54, 55), that took place between May and July 2018. The report was developed by Anvisa using the different proposals received, the scientific evidence available and international experiences on FoPNL. Anvisa’s preliminary proposal indicated WLs as the most adequate FoPNL for Brazil (Figure 2A).

Three days before the end of the TPS, ABIA presented a writ of mandamus (56) against Anvisa, asking for an extension of the consultation, so that the trade association could present its studies and tests in favor of the TLL. This strategy was used after Anvisa denied a request from *Rede Rotulagem* to extend the TPS until 24 July (57, 58). On that occasion, Anvisa appealed the decision and declared that there was “perplexity” regarding ABIA’s position (59). On July 11, 2017, the Federal Judge decided to defer ABIA’s request, which prolonged the TPS for 15 more days (for a process of a total of 60 days)–a decision justified by the soccer World Cup, an event during which Brazilian life gets disrupted, and a truck drivers’ strike, which also caused political challenges (56). The lawsuit of ABIA is a known CPA strategy that served to delay the process (Table 1).

In the TPS, ABIA called for the use of a modified design for the proposed TLL (Figure 1B), which was based on its work with external consultants hired by the trade association: nutrition researchers; companies undertaking research, design and communication, packaging, and economic analyses; and a law firm (60). The contributions of several food industry actors in the TPS were very similar to each other (even identical in some instances), signaling, at a minimum, some work in collaboration (a coalition management strategy) (20).

In July 2018, the consulting firm *GO Associados* published a non-peer-reviewed report entitled “Socioeconomic impacts of the implementation of nutrition labeling models on the front panels of foods and beverages,” commissioned by *Rede Rotulagem* (an information management strategy). The aim of the study was to estimate the socioeconomic impacts of the implementation of the TLL and the WL models. The results showed that there would be job and economic losses with the implementation of WLs: R$ 7.0 billion in production, more than 130,000 jobs, R$ 1.0 billion in payroll and R$ 617 million in taxes (61). These results were presented to Anvisa and disseminated in the media (62). The overestimations in the calculations and the lack of considerations about the potential positive impacts on population health of the adoption of WLs were ignored in that report (63).

Over the next few months, Anvisa changed its position regarding the appropriate FoPNL system, following a series of events. The former Director-President of Anvisa, Jarbas Barbosa, who was a supporter of the regulatory process on nutrition food labeling, left his office in July 2018. William Dib, one of Anvisa’s directors at that time, was nominated as Jarbas’ substitute by the former President of the Republic (2016–2018), Michel Temer (64) (Table 2). During a public lunch event of the Federation of Industries of the State of São Paulo (*Federação das Indústrias do Estado de São Paulo*–Fiesp), Temer indicated he was against WLs, arguing that this model was a “danger sign” that could “harm the (industry) sector.” At the same event, the former president of ABIA talked about the need to nominate a new Director-President of Anvisa who would be open to dialog with the industry (65). This is a key CPA strategy, a direct access and influence on the government. After Dib’s nomination as Anvisa’s Director-President in September 2018, he spoke in favor of the TLL, contradicting Anvisa’s own conclusions in its Preliminary Regulatory Impact Analysis Report on Nutrition Food Labeling (66).

In December 2018, Dib participated in the seminar “Right to information in food labeling,” hosted by *Valor Econômico*, an economics, business and finance newspaper. The event was supported by *Rede Rotulagem* and had speakers from ABIR, ABIA, CNI, the Brazilian Association of Nutrology (*Associação Brasileira de Nutrologia*–ABRAN), the Brazilian Society of Food and Nutrition (*Sociedade Brasileira de Alimentação e Nutrição*–SBAN) and PROTESTE, a non-profit consumers’ association (67).

In March 2019, Dib participated in another event of the food industry: the AnuFood 2019, the biggest food and beverage fair in Brazil. During a speech shared with the former president of ABIA, Wilson Mello (Table 2), Dib described the fair as “an opportunity for Anvisa to strengthen dialogs and issues and find interfaces,” meaning that the industry must be heard by the agency (a CPA strategy, with a direct contact and potential influence of the industry on government agencies). Dib also said that “social participation in the construction of the regulatory agenda is fundamental. It is essential for us at the agency that not only the consumer, but the regulated sector, participate in this process.” Dib was praised by Mello “for (his) always open, collaborative attitude” (68, 69).

A year after the launch of the TPS, in April 2019, Anvisa held a meeting to present the results of the contributions and the calendar with the next steps of the regulatory process. Next, in May (70), July (71), and August (72) 2019, Anvisa held three sectoral dialogs, technical meetings with the presentation of the official proposal of the agency for different aspects of the new nutrition food labeling regulation, based on the TPS contributions, international experiences and scientific evidence. Academia, civil society, food industry and government representatives participated in the discussions. The design and the NPM that would be chosen for the FoPNL were not presented or discussed on these occasions. A study developed by University of Brasília (*Universidade de Brasília*–UnB) on the performance and perception of five FoPNL systems was published in April 2020 and used by Anvisa. The study showed a better performance of the WLs when compared to the MG (the model presented in the PCs, not the one approved in 2020) and the TLL.

With the end of the sectoral dialogs at Anvisa, in September 2019, the agency published the Regulatory Impact Analysis Report on Nutrition Food Labeling (73) and opened the public consultations (PCs) no 707 (74) and no 708 (75), with a proposal for the new nutrition labeling regulation. Anvisa’s chosen FoPNL model was a MG that would indicate high amounts of added sugar, saturated fat and sodium, based on a two-step NPM developed by the agency (Figure 2B).

The PCs were supposed to last 45 days. However, after a request of ABPA, an entity that represents the poultry and pork meats sectors in Brazil and member of *Rede Rotulagem*, Anvisa extended the PCs for 30 more days, until December 9, 2019 (76). ABPA justification for the request was the need for more time to develop studies about the animal products’ labels, especially regarding their readability, considering that these products are under both Anvisa’ and the Ministry of Agriculture, Livestock and Supply (*Ministério da Agricultura, Pecuária e Abastecimento*–MAPA)’s regulation and that many of these products have common label for both national and international markets (77). This request represented another CPA strategy to delay the regulatory process, once more postponing the official timelines (Table 1).

The researchers from UnB (78) submitted a comment in the PC, arguing that the results of their study were not conclusive about the MG model, and said they were supportive of WLs instead (79). ABIA’s contribution to the PCs included another modification on its proposal for the FoPNL, with the reduction of the size of the MG proposed by Anvisa (Figure 1C).

Anvisa then proposed a new MG, since the WLs generate “fear,” a point which was noted in the study from UnB, and claimed in a speech of the former General-Manager of Foods at Anvisa, Thalita Lima (Table 2).

In 2019, it was reported by an investigative journalism platform that 90% of the meetings and events between Anvisa and outside organizations during that year were with companies and trade associations. The food industry was in second place in terms of numbers of meetings and events, behind the pharmaceutical industry (80). Lobbying activities are key CPA strategies used by the food industry to influence political decisions (Table 1).

In March 2020, a scientific paper led by the Brazilian Agricultural Research Corporation (*Empresa Brasileira de Pesquisa Agropecuária*–Embrapa) (81) (Table 2) compared the efficacy of different WLs, the MG (presented by Anvisa during the PCs), the TLL and the Guideline Daily Amounts (GDA). The study showed that WLs were the model with the best performance on all the evaluated aspects, and the use of familiar warning signs and the black color improved the efficacy of the FoPNL.

Anvisa soon had to face the COVID-19 pandemic from March 2020. This resulted in a delay to publish its decision about the new nutrition food labeling regulation. The food industry therefore asked for more *vacatio legis*^1^ time, from 12 to 24 months (82).

In June 2020, Italian food companies shared their criticism of the proposed FoPNL, through the Embassy of Italy in Brazil in a letter sent to the Presidency of the Republic and to Anvisa. A key argument was that WLs “look like symbols of danger” and “generate fear in consumers, demonizing entire food categories as they disregard the importance of recommended portions for each.” The letter mentioned that the Italian manufacturing sector had a great cultural importance in Brazil and that the food and beverage sector represented 9.6% of the gross domestic product (GDP) in the country, hence implying that any impact on the Italian food industry would have repercussions in Brazil. The letter also made it clear that WLs “attack the freedom of the consumers” (83) (Tables 1, 2). Those are arguments described in the CPA literature, when the food industry promotes its economic importance, which could refrain government from adopting too restrictive measures, even if at a cost in terms of population health.

Moreover, in 2020 Anvisa canceled its Collegiate Board (*Diretoria Colegiada*–Dicol) meetings and ran out of quorum of directors to vote for the approval and publication of its norms (84). After almost a year since the launch of the PCs, with no further advances with the FoPNL process, the Brazilian Institute for Consumer Defense (*Instituto Brasileiro de Defesa do Consumidor*–Idec), a non-profit organization and the main civil society organization involved in the nutrition labeling regulatory process (Table 2), presented a writ of mandamus to the Federal Supreme Court (*Supremo Tribunal Federal*–STF) against Anvisa’s Dicol, its President and the Presidency of the Republic. Idec requested for the appointment of new directors at Anvisa, and the inclusion of the regulatory process of the nutrition labeling of packaged foods on the agenda of the next Dicol meeting (85).

Anvisa’s new directors were later on appointed and, on October 8, 2020, the new regulation on nutrition labeling of packaged foods was approved unanimously. The approved Resolution of the Collegiate Board (*Resolução da Diretoria Colegiada*–RDC) no 429/2020 (16) and Normative Instruction (*Instrução Normativa*–IN) no 75/2020 (17) had yet a different version of the FoPNL (Figure 2C). The MG was reduced in size, occupying less space on food packages, thus being less easily readable, less clear and simple (86). This new model was never evaluated before and was not based on any scientific evidence of its effectiveness, but was closer to the design advocated for by the food industry. The NPM was also weakened, with a larger proportion of food products being exempt from carrying the MG, because their cut offs for nutrients of concern were increased. The use of nutrition claims was only limited to those nutrients present on the FoPNL; claims for other nutrients, such as vitamins, minerals and fiber, being therefore allowed. Finally, the *vacatio legis* time was extended for another 24 months, at the request of the food industry, and the norm will be implemented from October 2022, with an extension of three more years for reusable packaging such as soft drinks. The food industry was therefore, on numerous occasions, able to delay the process.

Right after the approval of the regulation by Anvisa, *Rede Rotulagem* published its position about the new norm: “Although the productive sector has defended a more informative model, having even suggested, in the PCs process, the TLL design (colorful GDA), we are confident that the model approved by Anvisa meets the proposed objectives since the beginning of the regulatory process.” The food industry also committed to undertake educational actions with consumers to guide them about the reading and understanding of the labels (87). This is a discursive strategy of the food industry, where it wants to be seen as a key actor in nutrition, and supports education so that individuals would be blamed if they continue to be sick, instead of the industry being questioned about the healthiness of its products.

In December 2020 (88), the General-Manager of Foods at Anvisa at that time, Thalita Lima, gave an interview to “ILSI in Focus” (*ILSI em Foco*, in Portuguese) (89), a *newsletter* of ILSI Brasil (International Life Sciences Institute), a well-known front group funded by and close to the food industry (89) (Table 2). When asked about the way that Anvisa had been contributing to the advances of ILSI Brasil, Thalita answered: “I understand that there is a symbiotic relationship between the agency and ILSI Brasil, in which both institutions benefit from working together. Anvisa, with its agenda of priorities, signals to the society which themes need to be the object of research and studies. On the other hand, organizations such as ILSI help to fill this gap, providing the agency with important scientific information for decision-making.” Lima also highlighted that “ILSI has historically contributed to various processes and activities of the agency. I would like to highlight the Institute’s participation in the meetings of the Codex Alimentarius Working Group on Nutrition and Food for Special Purposes and in the regulatory process for Food Supplements, which represented an important advance for the productive sector and for the citizen, with improved quality, safety and product effectiveness” (Table 1). This is an information management strategy, discussed in other studies of the CPA of the food industry (23).

Anvisa’s director and the rapporteur of the regulatory process on nutrition labeling of packaged foods, Alessandra Soares (Table 2), left its board in July 2021, and in the same month took up a position at Tavares Intellectual Property (Tavares Propriedade Intelectual, in Portuguese), which “advise clients on all matters related to the updated regulatory of foods and medicines, including, in particular, the acquisition of rights related to the registration of products before the main regulatory bodies” (90) (Table 1), a CPA practice known as the “revolving door,” with a former employee goes with its knowledge and relationships from the government to the industry.

In the second semester of 2021, Anvisa launched a series of actions, with the publication of “Questions and answers about nutrition labeling of packaged foods” (91), and, in December, two webinars to present the new regulation and to answer questions about the FoPNL (92) and the nutrition facts panel (93).

In September 2021, a scientific paper written by Unilever’s employees (Table 2) about the comparison of the Nutri-Score, another hybrid label, ABIA’s model, Idec’s model and Anvisa’s model (the MG proposed in the PCs and not the one approved in 2020). The paper discussed the potential of each model to help individuals make healthier food choices. Nutri-Score is a color-coded nutrient profiling system (21), adopted voluntarily by the food industry in some European countries (94), and supported by WHO Europe and some European Union (EU) countries to be mandatorily adopted in the EU. Nutri-Score was subject to intense criticism and lobbying from the food industry, as described elsewhere (21, 95, 96). It is however now accepted and even supported by food companies, and preferred to the WLs. When compared to the control regarding the usefulness of each model to make healthier choices, the hybrid label and the ABIA’s model performed best, followed by Nutri-Score. Idec’s and Anvisa’s models performed worse (97). The conclusion of the paper, based only on subjective measures about the perception of consumers, favored the model supported by ABIA and its members, including Unilever (Table 1).

In March 2022, the platform *Olho na Lupa*, described as “an initiative of 11 associations linked to the food and beverage industry and retail that aims to inform and educate Brazilian consumers about the new food labeling,” was launched. The initiative has an official website (30) and social media profiles (98–101) and has ABIR, ABIA, ABPA, ABRAS, *Viva Lácteos* and CNI among its members (Table 2). The content of *Olho na Lupa* is based on the new regulation of nutrition labeling on packaged food. Regarding the FoPNL’s NPM, the initiative explains that “Any food can be part of a healthy diet. With the indications of the nutrition label, you have more information to make your choices and compose a diet that is most appropriate to your needs and preferences,” thus once again arguing that individuals are responsible for their own choices, and being silent on the fact that the consumption of certain products lead to ill-health (Table 1).

In April 2022, a scientific paper comparing the effectiveness of the FoPNL designs in the format of triangles, as proposed by civil society organizations, and the MG, as proposed by Anvisa in the PCs in 2019, was published. According to the participants of the study, the triangular model communicated important information, was a useful tool and was easier to understand. However, both models performed similarly in communicating nutrient information and the MG model performed marginally better at improving purchase intentions (102). This study was developed by researchers from the Center for Epidemiological Research in Nutrition and Health/University of São Paulo (*Núcleo de Pesquisas Epidemiológicas em Nutrição e Saúde/Universidade de São Paulo*–Nupens/USP) (Table 2) and Idec and used the MG presented in the PCs by Anvisa and not the one approved in 2020, which was not tested yet.

Throughout the entire period of the regulatory process in Brazil, food labeling was also being discussed in MERCOSUR and in Codex Alimentarius. In both spaces there is the active participation of Brazilian food industry’s and civil society’s representatives in the internal meetings with Anvisa to discuss the Brazilian position, and as observers in the international meetings when each country defends its position. This might have led to influence from the food industry at the regional level.

## Discussion

Throughout the regulatory process for the adoption of a new nutrition labeling of packaged foods in Brazil, the food industry used various CPA strategies, and different situations of CoI situations were identified. The results of this study add evidence to the existing literature in that space (21, 24) and, particularly, on the efforts of industry actors to negatively influence the development of new FoPNLs (20, 22, 23, 103).

The food industry used “discursive” strategies in its presentation of commissioned reports and studies. Other organizations and individuals close to the industry also used arguments similar to the food industry such as ABRAN, the Embassy of Italy in Brazil and even a former leader of Anvisa. The main tactic of the food industry consisted in framing the debate, specially shifting the blame away from the food industry and its products in the ill-health of the population, and promoting industry’s preferred solutions such as balanced diets and education, instead of a label that would be too restrictive for their products. The economy and the expected food industry costs were also mentioned, specially the potential losses in jobs and sales (20), argument also used against a proposal to reduce tax incentives for producers of sugary drinks in Brazil (104). These arguments were used before and during other regulatory processes on food labeling in Latin America (22, 23, 105, 106).

The main CPA action used by the food industry in addition to these arguments was its “direct involvement and influence in policy.” Through the provision of technical support and advice to policymakers and lobbying, the food industry participated in technical discussions, PCs and meetings with decision makers, with no questioning about its conflict in defending commercial interests. Industry participation was also reported during the Mexican discussions about FoPNL, with a mandatory, biased and disproportional presence of the food industry representatives (105). Food industry representatives had access to Anvisa’s technical and political leaders, with much more opportunities than civil society and academia. This helped the food industry present its arguments, lobby and influence the decisions regarding the nutrition labeling regulation, a situation that was observed in other contexts in Brazil (104). Even when the decision for a new FoPNL system was reached, high ranking individuals in Anvisa used the revolving door, which may be a strategic practice for the food industry in the future.

The “coalition management” was used to establish relationships with key opinion leaders, such as influencers, health organizations, and Anvisa’s leaders. The establishment of relationships with the media also occurred in order to disseminate the food industry’s narrative among journalists and, consequently, to the general population, a strategy that was already reported in previous analysis of the influence of the food industry in Brazil (104). Through the creation of *Rede Rotulagem*, the food industry managed to reach different audiences and to strengthen its voice in the regulatory process, a well-known strategy for corporations to avoid being in the spotlight. This strategy was also used in Colombia, while the FoPNL policy was being discussed in Congress (23). Eleven of the same organizations that were members of *Rede Rotulagem* created another initiative, *Olho na Lupa*, six months before the beginning of the implementation of the regulation, for helping consumers read and understand the food labels, reinforcing the idea that individuals are responsible for their own health. In this way, the food industry puts itself as part of the regulatory process and as defender of Anvisa’s regulatory decision.

The “information management” strategy was mainly observed through the studies commissioned by the food industry, with the production and amplification of information that supported its narrative. These studies were not peer-reviewed and were produced by hired consultants or companies related to research, nutrition, design and law. Using that information, the food industry managed to disseminate its arguments related to its support for the free will of individuals, the need for more education and information, the risks of economic losses, the discrediting of WLs, and the superiority of TLLs. That last point was particularly stressed during the beginning of the FoPNL discussions.

Finally, “legal actions” were used once in the beginning of the regulatory process, when an informal request by the food industry to Anvisa was not sufficient to extend the TPS. It was a strategic move since Anvisa had first discussed its preference for WLs. In order to postpone the advance of the regulatory process, the food industry presented a lawsuit and then earned more time. ABPA’s request later during the could have turned into another lawsuit against Anvisa if the agency did not agree with the extension of the deadline to present the contributions. A lawsuit was previously used by the food industry to prevent the implementation of a policy focused on childhood marketing restriction in Brazil (23).

**FIGURE 3:**
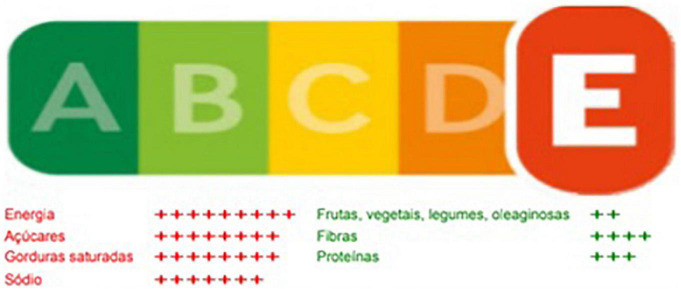
Front-of-package nutrition labeling (FoPNL) model proposed by ABRAN. Source: ABRAN.

It is important to highlight that the FoPNL process was not only influenced by the actions and discourse of the food industry itself, but also by other actors, some of which have closer ties to the companies, and others less so. ABRAN is one example. In February 2018, this association, which had not been part of the WG on nutrition food labeling, sent an adapted version of the Nutri-Score FoPNL as a proposal to Anvisa (Figure 3). After the publication of the Anvisa’s Preliminary Regulatory Impact Analysis Report, which indicated WLs as the most adequate FoPNL for Brazil, in June 2018, ABRAN requested the agency to reconsider its model, and use instead an adaptation of the Nutri-Score (107). This request was made after the former Ministry of Health, Gilberto Occhi, praised the Nutri-Score model after a trip to Europe, also citing the TLL, both models already discarded by Anvisa at that time. ABRAN is an association that maintains partnerships with the private sector both in the organization of the Brazilian Congress of Nutrology (108) and in the production of scientific materials and that has already been criticized for its CoI situations related to food manufacturers (109). The researcher responsible for the adaptation of the Nutri-Score had CoI: he was lead author of a Danone-funded article, and participated in a Nestlé event for the promotion of growing-up milks (110). He also coordinated a webinar of ILSI Brasil and is a member of its Scientific Committee–the webinar was focused on the Danone-funded article (111).

Another example of action not taken by the industry itself, but by other organizations and institutions using similar arguments and apparently converging interests, was the case of the Embassy of Italy in Brazil. The connection between the country’s official representatives and food companies, especially Ferrero, the Italian transnational manufacturer of confectionery products, are not new. A former advisor of the Ferrero group was a member of Italy’s Foreign Relations delegation and responsible for Italy’s position against recommendations of WHO on sugar intake in 2015 (112, 113).

Our results are similar to the existing evidence about the use of CPA strategies by the food industry during FoPNL discussions in other countries of the region, such as Uruguay, Colombia, Mexico, Argentina, and the Caribbean (22, 23, 105, 106). This could be explained by the fact that many of the actors included in this study are transnationals (or associations representing the transnational companies) or belong to international organizations, such as ILSI. Considering regional economic blocs, like MERCOSUR, the argument that a county needs harmonization before a country could introduce a FoPNL national policy was used in Argentina to try to delay internal discussions. In Jamaica, as the Caribbean Community (*Comunidad del Caribe*, in Spanish) was debating the adoption of a FoPNL, a local lobby organization representing the manufacturing and ultra-processed food products sectors argued that WLs do not align with the realities of Jamaica’s major trade partners, which, together with the use of other CPA strategies, resulted in the rejection of the proposed WLs (106).

Civil society and academia have actively participated in the Brazilian regulatory process on nutrition labeling since its inception with strategic actions that strengthened the process for approval of the FoPNL such as: mobilization campaigns in social media, mass media campaigns and petitions, propositions and participation in public hearings, carrying out FoPNL activities at events with medical societies, at universities, in schools, and communities. Those actors took part in the entire regulatory process, including the TPS, the PCs and meetings with Anvisa’s technical and political leaders. These actions were led by Idec and the Alliance for Adequate and Healthy Diets (*Aliança pela Alimentação Adequada e Saudável*, in Portuguese) (114) (Table 2). The collaboration of academia was crucial to provide the scientific bases for the process.

The process analyzed here, led by Anvisa, allowed for the participation of civil society, academic and food industry representatives. This is also the case for other instances. Codex Alimentarius and MERCOSUR, for example, are spaces with stimulated and permitted participation of the food industry together with other actors. However, it is important to highlight that this participation is not balanced, with more representatives from the food industry than civil society, which could lead to a bias in the voices heard and influence the final position of countries. This situation is especially worrying because of the importance of these international spaces and their influence on countries. Codex is a program of the Food and Agriculture Organization of the United Nations (FAO) and WHO to set standards and guidelines for food regulation (115). Despite being a recommendation, Codex is usually used by the food industry as an attempt to delay national or regional discussions (23). MERCOSUR is a regional integration space to facilitate trade and investment between its country members (116). Some of the country’s representatives are from the economy and trade sector of the government, and not all the countries have civil society representatives actively participating in the process, which means that public health and consumers’ rights might not be prioritized during the discussions. Codex and MERCOSUR are both platforms through which the industry gains access to decision-making, which could be at the detriment of public health by establishing weak and corporate-friendly standards and agreements (117).

It is worth mentioning our study’s limitations and strengths. We did not use interviews as a way to collect or triangulate information about the policy process. To overcome this gap, the data collection included the use of the LAI, besides publicly available information. Furthermore, many of this article’s authors have direct involvement in the topic under study as representatives of civil society and investigative journalists, which represent a bias toward public health, rather than economic interests.

This is an original work that brings together the analysis of the CPA and CoI situations and the record of the regulatory process of the approval of a new FoPNL in Brazil. Since Latin America and the Caribbean countries are advancing in FoPNL regulation, this work is especially important to inspire them, document and share learnings and experiences about the food industry interference and possible ways to overcome these barriers. This paper was only possible to be developed because of the existing evidence and documentation of the regulatory process produced and disclosed by the civil society, the academia and the media in Brazil.

The analysis of the regulatory process for the adoption of the new nutrition labeling of packaged foods in Brazil demonstrated various CPA and CoI situations involving the food industry, which had a negative impact in the regulatory process, leading it to an approved FoPNL regulation which was neither aligned with the recommendations of international health organizations, nor with existing independent scientific evidence, nor the region’s most recent experiences. The approval of a MG with a flexible NPM and a long adaptation period reflects the requests of the food industry.

In order to have the most adequate FoPNL regulation as possible, it is important to protect the process of the food industry interference by having mechanisms to avoid CoI. In this regard, PAHO recently launched a CoI prevention tool entitled “Prevention and management of CoI in nutrition programs at the national level” is promising (118). It presents a step by step of how governments and health ministries should proceed before establishing a relationship with non-state actors, investigating the actor’s alignment, the profile of the interaction, and also the assessment of risks and benefits of the interaction. The implementation of this type of tool would allow greater protection of the political process, so that the primary interest of the policies prevails. It should be used to inspire other countries that are still in the process of formulating and discussing nutrition labeling standards.

Even so, from October 2022 Brazilian consumers will have more explicit information about the high amounts of nutrients of concern for health in food labels, which may help them make healthier food choices and improve health and prevent obesity and NCDs.

## Data availability statement

The raw data supporting the conclusions of this article will be made available by the authors, without undue reservation.

## Author contributions

LAM, MM, and CMPC: conceptualization. LAM, MM, BKH, and CMPC: methodology. LAM, BKH, JMDP, MGS, and CMPC: writing—original draft preparation. LAM, MM, BKH, JMDP, MGS, APBM, JGC, and CMPC: writing—review and editing. LAM and CMPC: project administration. APBM and JGC: funding acquisition. All authors contributed to the article and approved the submitted version.

## Footnotes

1 Latin expression that means the period between the date of publication of a legal norm and the beginning of its validity. Generally, the vacatio legis is expressed in an article at the end of the law with the following text: “This law enters into force after (the number of) days of its official publication.”
